# Development and testing of a text messaging (SMS) monitoring software application for acute decompensated heart failure patients*

**DOI:** 10.1590/1518-8345.3519.3301

**Published:** 2020-09-07

**Authors:** Leticia Lopez Pedraza, João Ricardo Wagner de Moraes, Eneida Rejane Rabelo-Silva

**Affiliations:** 1 Universidade Federal do Rio Grande do Sul, Porto Alegre, RS, Brazil.; 2 Hospital de Clínicas de Porto Alegre, Serviço de Cardiologia, Grupo de Insuficiência Cardíaca e Transplante Cardíaco, Porto Alegre, RS, Brazil.; 3 Universidade Federal do Rio Grande do Sul, Escola de Enfermagem, Departamento de Enfermagem Médico-Cirúrgica, Porto Alegre, RS, Brazil.

**Keywords:** Telemedicine, Heart Failure, Medical Informatics, Monitoring, Text Messaging, Nursing Education, Telemedicina, Insuficiencia Cardíaca, Informática Médica, Monitorização, Mensagem de Texto, Educação em Enfermagem, Telemedicina, Insuficiência Cardíaca, Informática Médica, Monitoreo, Mensaje de Texto, Educación en Enfermería

## Abstract

**Objective:**

to develop and test an SMS monitoring software application for patients with acute decompensated heart failure.

**Method:**

the waterfall model was used for software development. All expected functionalities were defined, program modules were codified and tests were done so as to ensure good performance by the software application. Ten patients participated in the prototype test.

**Results:**

the system sends two types of messages: questions that should be answered by patients and unilateral educational reinforcements. In addition, the system generates alarms in case of no response or according to a flow chart to detect congestion in the patient previously created by the team. Of the 264 SMS texts sent, 247 were answered. The alarm was triggered seven times: three patients woke up with shortness of breath for two consecutive nights, and four patients felt more fatigued for two consecutive days. All patients took the prescribed medications during follow-up. The study nurse guided the patients who generated alarms in the system.

**Conclusion:**

the SMS software application was successfully developed and a high response rate and preliminary evidence of improvements in self-management of HF were observed. With this regard, telehealth is a promising alternative in the treatment of chronic diseases

## Abstract (pt)

**Objetivo:**

desenvolver e testar um *software* de monitoramento de SMS para pacientes com insuficiência cardíaca aguda descompensada.

**Método:**

o modelo em cascata foi utilizado para o desenvolvimento do *software*. Todas as funcionalidades do software foram definidas, os módulos do programa foram codificados e os testes foram realizados com o objetivo de assegurar bom desempenho. Dez pacientes participaram do teste do protótipo.

**Resultados:**

o sistema envia dois tipos de mensagens: perguntas que devem ser respondidas pelos pacientes e reforços educativos unilaterais. O sistema também aciona alarmes no caso de não haver resposta por parte do paciente ou de acordo com um fluxograma criado pela equipe para detectar congestão nos pacientes. Um total de 267 mensagens foram enviadas, das quais, 247 foram respondidas. O alarme foi ativado sete vezes: três pacientes acordaram com falta de ar por duas noites consecutivas e quatro pacientes se sentiram fatigados por dois dias consecutivos. Todos os pacientes tomaram suas medicações durante o seguimento. A enfermeira do estudo instruiu os pacientes que tiveram alarme.

**Conclusão:**

o *software* SMS foi desenvolvido com sucesso e teve alta taxa de resposta. Também obtivemos evidência preliminar indicando melhor autogestão de IC. Neste sentido, a telessaúde se apresenta como uma alternativa promissora no tratamento de doenças crônicas.

## Abstract (es)

**Objetivo:**

desarrollar y probar un *software* de monitoreo de SMS para pacientes con insuficiencia cardíaca descompensada aguda.

**Método:**

el modelo en cascada se utilizó para el desarrollo del *software*. Se definieron todas las funcionalidades esperadas, se codificaron los módulos del programa y se realizaron pruebas para garantizar un buen rendimiento del *software*. Diez pacientes participaron en la prueba del prototipo.

**Resultados:**

el sistema envía dos tipos de mensajes: preguntas que deben ser respondidas por los pacientes y refuerzos educativos unilaterales. Además, el sistema genera alarmas en caso de no respuesta o de acuerdo con un diagrama de flujo para detectar congestión en el paciente previamente creado por el equipo. De los 264 SMS enviados, 247 fueron respondidos. La alarma se activó siete veces: tres pacientes se despertaron con dificultad para respirar durante dos noches consecutivas y cuatro pacientes se sintieron más fatigados durante dos días consecutivos. Todos los pacientes tomaron los medicamentos recetados durante el seguimiento. La enfermera del estudio orientó a los pacientes que generaron alarma en el sistema.

**Conclusión:**

el *software* de SMS se desarrolló con éxito y se observó una alta tasa de respuesta y evidencia preliminar de mejoras en el autocontrol de la insuficiencia cardíaca. En este sentido, la telesalud parece ser una alternativa prometedora en el tratamiento de enfermedades crónicas.

## Introduction

Hospitalizations for heart failure (HF) in Brazilian public hospitals represent approximately 28% of all hospitalizations due to cardiovascular diseases and 2% of those for all other diseases^(^ 1 ^)^. In the elderly adult population, HF is already the main cause of hospitalization in the country. Another alarming rate is related to high mortality: it is estimated that from 2 to 17% of the patients admitted for HF will die while in hospital. However, for those treated in the outpatient setting, survival is greater because of stability^(^ 2 ^)^.

In this case, HF incidence was higher than that of other cardiovascular diseases^(^ 3 ^-^ 4 ^)^, resulting in high costs^(^ 5 ^)^. These costs are the sum of several components, including acute decompensation management, clinical consultations, medications, home care and the increasing cost of implantable devices^(6-8^. Despite all the advances in the care for patients with HF, results after hospitalization are still fewer than expected^(^ 9 ^)^. Strategies and new approaches are necessary in the current worldwide panorama of HF epidemiology, in relation to both hospital readmissions and the morbidities caused by this clinical syndrome^(^ 10 ^-^ 11 ^)^.

It is in this scenario that we can use the beneficial results of recent studies showing that telehealth can be a promising alternative in the management of chronic diseases^(^ 12 ^-^ 14 ^)^. In Brazil, monitoring and education programs involving complex methodologies and technologies could have limited practical applicability, taking into account the social, economic and cultural peculiarities of the country.

Mobile technology, in particular the Short Message Service (SMS), is emerging as a promising platform for chronic disease management in low-income populations^(^ 15 ^-^ 17 ^))^ because it has high rates of utilization across socioeconomic groups^(^ 18 ^-^ 20 ^)^. Public health researchers have sought to capitalize on this potentially game-changing communication modality by developing and testing SMS interventions designed to provide information that results in improved health outcomes and/or changed health behaviors. In slightly more than a decade of innovative research, dozens of studies and more than 20 systematic reviews and meta-analyses have been conducted to explore the potential of SMS for public health^(^ 21 ^)^.

Given this scenario, SMS seems to be a promising possibility because it is a simple and low-cost technology that facilitates real-time and individualized monitoring of patients. Telemonitoring HF patients will prevent or reduce congestive symptoms, as well as help the early identification of clinical deterioration signs through a rigorous system of monitoring such signs and symptoms^(^ 22 ^-^ 23 ^)^. Thus, this technology will enable the early detection of HF decompensation through real-time intervention, achieving better results and reducing costs in the health system^(^ 24 ^)^.

It is not known that an SMS prototype has been developed as a monitoring strategy for patients with HF and recent hospitalizations in a public university hospital. Aiming to fill this gap, the present study was conducted in order to test a monitoring prototype for patients with HF through SMS.

## Method

This is a study on the development of a remote monitoring software application for patients with acutely decompensated HF.

The proposed system is a patient-interface solution based on the exchange of SMS-type messages combining the mobility features found in mobile applications and the simplicity and greater accessibility of the population provided by SMS technology. The software application allows sending previously developed questions with positive or negative answers or numerical values and enables the analysis of such responses in real time.

The waterfall model was used for software development:

1) Specification: The purpose of this step was to define, in a detailed way, all the functionalities expected to be contained in the prototype. This stage was of fundamental importance to enable agile and low rework-rate development. There were several meetings with the multiprofessional team to determine the content and number of messages.

2) Development: The purpose of this stage was to encode the software modules so that all previously defined specifications were met. The system has four modules:

- Graphic interface: It is the form of communication between the user and the software. This module comprises the development of the entire system user interface, such as form fields, buttons, graphic generations and other graphic elements that are needed (Figure 1).
  **Figure 1:**
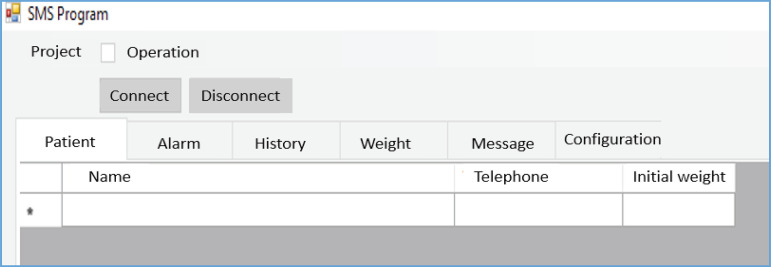
Interface
- Database: It stores different information - list of patients and their details, event records, among others.
- It sends and receives SMS messages.
Functionalities: the system’s functionalities are the result of the integration of the previously described modules, which were divided into those automatically executed by the system and those executed through commands by an operator.

The automatically executed features allow monitoring data records and performing the checks for all users using a flowchart previously created by the team. These checks are “tendency to gain or lose weight” and “lack of response”, among others. If such a tendency is identified, the system generates an alarm and sends information to the patient as well as to the person in charge of managing the system in the hospital where it is being monitored.

Through the functions executed by commands, the professional can use certain features, such as user addition/editing, patient history visualization and alarms.

3) Verification: based on the performance of different types of tests, including performance tests and operating limits.

**Figure 2:**
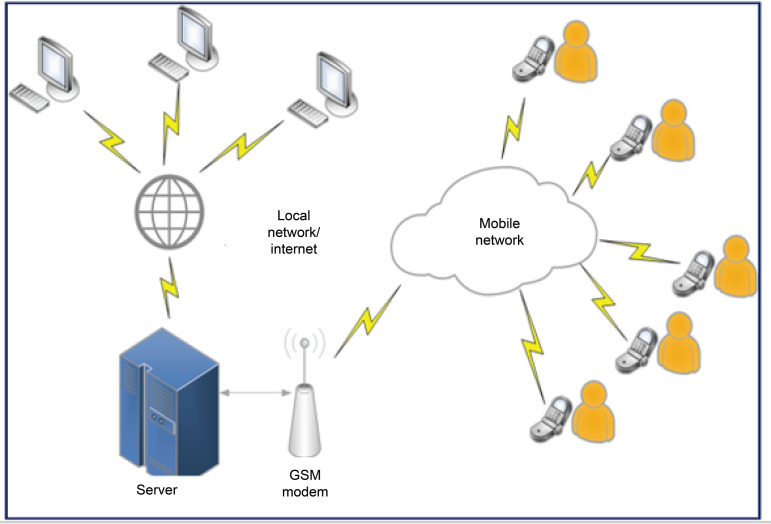
Infrastructure diagram of the proposed system

In order to be able to use the system simultaneously in different places, it runs on a server, and access to it is obtained via the web. Thus, it allows the gain of scale in its use without the need for specific software tools to the work machines located in hospitals. At the same time, the system limits access to patients in a specific hospital in order to ensure privacy of information. The infrastructure of the proposed system is shown in Figure 2.

The main components of the system architecture are:

- Machines: Conventional computers with Internet and/or intranet connection. They do not require specific tools for use.
- Server: Device responsible for executing the system logic, database and interface with a Global System Modem for Mobile Communications (GSM).
- GSM modem: Equipment for sending and receiving SMS-type messages.
- Patient’s mobile phone: Patient interface for receiving and sending SMS messages. There is no specific requirement for operating systems, makes or models of devices. There must only be SMS support and the basic functionality of all devices marketed in the last 10 years.

*Data collection and selection of participants for the test study:* We studied the use of the software application for a three-month-period, starting in September 2017. All patients were adults of both sexes with an HF diagnosis, regardless of its etiology, and left ventricular ejection fraction (LVEF) ≤ 45%, literate (or who had a literate caregiver), with a telephone available for post-discharge contact. Patients who had neurological or cognitive sequelae that prevented them from giving message feedback and those institutionalized were excluded; however, patients who had a relative or caregiver without the two former conditions could enter the study.

The software application and logistics test proposed by this development study were planned. Ten patients were invited to participate in the test. After acceptance by the patient during his/her hospitalization, all guidelines were provided, and doubts were addressed in subsequent visits. At this stage, the patient received a care manual with the most important information concerning the prototype. According to the literature, patients’ hospital readmissions are more frequent during the first few weeks after discharge^(^ 25 ^)^. For this reason, the patients received the message during the first week after discharge.

The program developed sent two types of messages:

- Feedback: Questions that should be answered by patients. The system sent two messages in the morning and two at night.
  - “During last night, did you wake up with shortness of breath once?” - YES or NO
  - “What’s your weight today?” - The patient/caregiver - Kg
  - “Have you eaten all your meals today?” - The patient/caregiver -YES or NO
  - “Have you felt more tired today than you did yesterday?” - YES or NO
- Educational: they did not require an answer. The system sent a message every two days.
  - Avoid processed meats because they all contain a lot of salt.
  - Shortness of breath is one of the symptoms caused by the accumulation of fluid in the body.
  - It is important for patients with heart failure to weigh themselves regularly at the same time each day and record their weight.

An important characteristic of the software application developed is related to its features, which are executed automatically and enables the hospital computer to receive alarms in the following cases: a patient’s not responding for two consecutive days; an affirmative answer to the question “During last night, did you wake up with shortness of breath?” for two consecutive days; two Kg of patient weight increase in three consecutive days; a negative answer to the question “Have you taken all your medications today?” for two consecutive days; and an affirmative answer to the question “Have you felt more tired today than you did yesterday?” for two consecutive days.

Descriptive data were processed and analyzed using the Statistical Package for Social Science (SPSS), version 18.0 for Windows. Categorical variables were summarized as frequencies and percentages, and continuous variables as means and standard deviations. 

This study was approved by the Ethics Committee of the *Hospital de Clínicas de Porto Alegre*, with CAEE number 62429916.3.0000.5327 and GPPG 160620, and all patients signed an Informed Consent Form (ICF) before entering the study.

## Results

**Figure 3:**
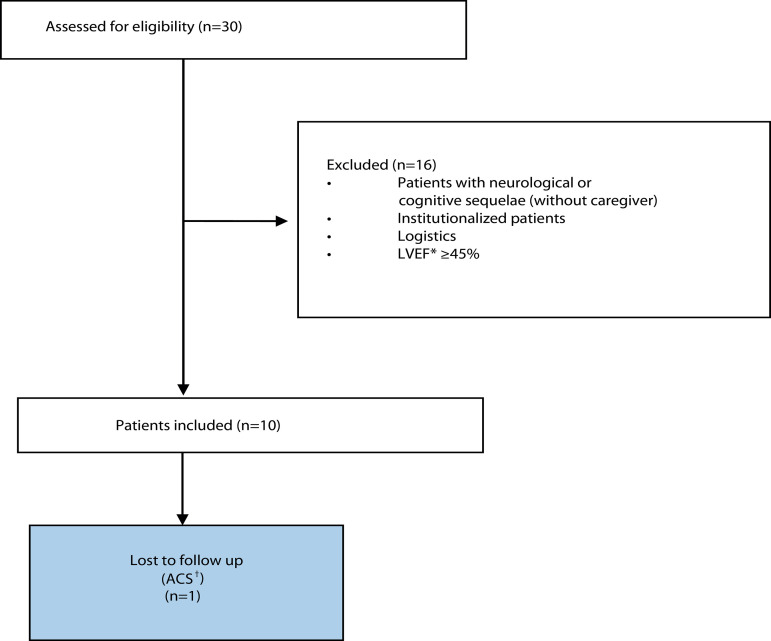
Patient flow diagram

The approximate time for the software application development was six months. Of the 30 eligible patients, 10 were included in the prototype test. One of them did not complete the seven-day follow-up because he was re-hospitalized for acute coronary syndrome. Of the 264 SMS messages sent, 247 were answered. Ten of the unanswered messages coincided with the lack of electric power generated by climatic conditions. The other messages were not answered because the patients did not see them (four) or because they forgot (three) to do it. The alarm was triggered seven times: three patients woke up with shortness of breath for two consecutive nights, and four patients felt more fatigued for two consecutive days. No patient gained two Kg in weight in three days. All patients took the prescribed medications during the follow-up. The study nurse guided the patients who generated alarms in the system (Figures 3 and 4).

*LVEF = Left ventricular ejection fraction; ^†^ACS =Acute coronary syndrome

**Figure 4:** Results

**Did you wake up during the night with shortness of breath?**	**Have you taken all the medications today?**
• 8 patients: 7/7 SMS* messages answered • 1 patient: 2/7 SMS messages answered • 1 patient: 3/3 SMS messages answered (ACS^†^) - 3 patients answered "yes" on two consecutive days	• 7 patients: 7/7 SMS messages answered • 1 patient: 6/7 SMS messages answered • 1 patient: 3/7 SMS messages answered • 1 patient: 3/3 SMS messages answered (ACS^†^) - All answers were affirmative
**What is your weight today?**	**Have you felt more tired today than you did yesterday?**
• 8 patients: 7/7 SMS messages answered • 1 patient: 3/7 SMS messages answered • 1 patient: 3/3 SMS messages answered (ACS^†^) - No patient increased 2kg in 3 days	• 7 patients: 7/7 SMS messages answered • 1 patient: 6/7 SMS messages answered • 1 patient: 5/7 SMS messages answered • 1 patient: 3/3 SMS messages answered (ACS^†^) - 4 patients answered "yes" for two consecutive days

* SMS = Short message service;

† ACS =Acute coronary syndrome

The patients’ mean age was 67±13 years. They were predominantly males and lived with relatives. Their mean ejection fraction was 35±7%. Table 1 illustrates sociodemographic and clinical characteristics.

**Table 1:** Patients' sociodemographic and clinical characteristics, Porto Alegre, RS, Brazil, 2017

Variable	Total n=10
Age*	67 ± 13
Sex, male^†^	8(80)
Living with family^†^	7(70)
Hospital stay^‡^	7(5-22)
Professional status, inactive^†^	6 (60)
Number of medications >5^†^	9 (90)
Ejection fraction*	35 ± 7

* Mean and standard deviation (SD);

† n (%);

‡ Median and interquartile range (25%-75%)

## Discussion

This study enabled the development of a prototype for remote monitoring through SMS. The testing of its functioning proved it to be adequate for the proposal of returning answers to questions and educational-reinforcement information, as well as to alarms.

During the test, the response rate was high and the patients’ statements showed that the SMS technology helped with the management of the syndrome. Participants reported no problems responding to SMS messages and described that system simplicity was essential for their adherence to the test. Many reported that they would continue being monitored because they felt that health professionals were with them at home.

Mobile telephone technology is emerging as a promising platform for chronic disease management^(^ 26 ^-^ 27 ^)^. Recent studies on telemonitoring interventions in patients with HF have been successful in reducing hospital readmissions^(^ 28 ^)^. However, such interventions require Internet- or Bluetooth-enabled phones, which are not always available to low-income populations.

SMS seems to be a promising alternative, considering the social, economic and cultural peculiarities of the population served in public hospitals in Brazil. Short message intervention has shown benefits in facilitating self-care of chronic diseases in Asian and African countries^(^ 29 ^)^. Studies on diabetes using SMS as a means of education or bi-directional communication have shown significant improvement in glycemic control^(^ 29 ^-^ 30 ^)^. In another study in Kenya, patients under antiretroviral treatment received weekly support from nurses’ text messages, resulting in improved adherence to treatment and suppression of plasma HIV-1 RNA load^(^ 31 ^)^. Recent studies have developed tools for monitoring patients through SMS^(^ 32 ^)^. This technology was well accepted by patients as both a reminder and an educational method.

In this context, the developed prototype adds an additional functionality, being able to generate alarms according to a predetermined flowchart. It is not of our knowledge that the latter functionality is being developed for patients with HF and recent hospitalization in the context of global health.

Technology-based approaches have the potential to reach a relatively high number of people from any population at risk. This can have a substantial impact on public health even when the effects are modest^(^ 33 ^)^. With help from technologies, patients can feel more motivated about their health by feeling as participants in their own care. Technologies can also favor their empowerment, implying a paradigm shift in the care of patients with chronic diseases. These results encourage us to conduct more robust studies using SMS technology as a tool for self-management of this syndrome - compared to other strategies, such as telephone monitoring or the usual care received at specialist treatment centers.

Our study has limitations. Firstly, over time, a difficulty arose in sending SMS messages. The telephone company blocked the sending of messages by the software application because it was considered to be a corporation that was sending SMS messages illegally. This happened because the same messages were sent at the same time every day. This situation should be resolved before beginning monitoring in future studies. Secondly, SMS messages sent as answers by patients have a cost, and it should be taken into account in future projects.

## Conclusion

In conclusion, a software application was developed successfully, and it is working properly. After testing, some changes, such as redefining the graphic interface, database and system engineering specifications, have been made to improve its operation. From the positive results in this study, it is considered that sending SMS messages can be a useful tool to improve self-management of HF by patients after hospital discharge. Although the software application works properly, further studies are needed to achieve significant results in clinical outcomes, such as morbidity and mortality.
